# Physiotherapy for Sexual Dysfunctions in Multiple Sclerosis Patients: A Review of Clinical Trials

**DOI:** 10.3390/jcm14103509

**Published:** 2025-05-16

**Authors:** Michalina Reimus, Mariusz Siemiński

**Affiliations:** 1 Emergency Department, University Clinical Centre, 80-214 Gdansk, Poland; mreimus@uck.gda.pl; 2 Department of Emergency Medicine, Medical University of Gdansk, 80-210 Gdansk, Poland

**Keywords:** multiple sclerosis, sexual dysfunctions, physiotherapy

## Abstract

Multiple Sclerosis (MS) is an inflammatory autoimmune disorder that primarily affects the central nervous system, leading to significant disability in young adults, with a higher prevalence observed in women. The heterogeneous clinical presentation of MS poses substantial challenges in diagnosis and treatment. In recent years, considerable emphasis has been placed on improving the quality of life for MS patients, with sexual health being a key component of this assessment. The literature suggests that sexual dysfunctions affect between 42% and 73% of individuals with MS. While the positive effects of physiotherapy interventions on sexual dysfunction have been extensively studied, there remains a significant gap in understanding the prevalence of these disorders within the MS population and the most effective treatment approaches. This review highlights the critical need to integrate pelvic floor physiotherapists into multidisciplinary MS treatment teams. Six randomized controlled trials (RCTs) meeting the inclusion criteria were identified. These studies included patients diagnosed with MS who reported various sexual dysfunctions, such as orgasmic dysfunction, sexual arousal disorders, and dysfunctions associated with overactive bladder. The outcomes were assessed using sexual function and urological symptom questionnaires. Physiotherapeutic interventions, including manual therapy, kinesiotherapy, and physical therapy elements, have demonstrated positive effects on sexual quality of life, specifically in relation to issues such as desire, arousal, and satisfaction. However, the available data are still preliminary and warrant further investigation. By recognizing the importance of pelvic floor therapy, more targeted and holistic interventions can be implemented, ultimately improving patient outcomes and enhancing the overall standard of care.

## 1. Introduction

### 1.1. Nature of Multiple Sclerosis

Multiple Sclerosis (MS) is an inflammatory auto-immunological disease of the central nervous system. It is one of the leading causes of disability in young adults, typically affecting individuals between the ages of 20 and 40, with a higher prevalence in women. The etiology of MS is multifactorial, involving both genetic predispositions and environmental factors such as viral infections [1]. The disease manifests through a wide range of neurological symptoms including vision problems, motor impairments, coordination issues, and cognitive dysfunction. Due to the heterogeneity of its clinical presentation and disease course, the diagnosis and treatment of MS presents significant challenges. Although there is currently no cure, advances in immunomodulatory therapies have significantly improved disease management, slowing progression and enhancing patients’ quality of life [2]. A deeper understanding of the pathophysiology of MS is essential for the development of more effective therapeutic strategies.

### 1.2. Epidemiology of Multiple Sclerosis

It is currently estimated that around 2.8 million people worldwide have MS, although these figures may be unreliable due to differences in diagnostic practices and patient reporting of symptoms in regions with limited access to healthcare. The prevalence of MS shows significant geographic, demographic, and ethnic variation [3,4]. The highest rates of incidence are observed in countries with temperate climates, particularly in Northern Europe, North America, and Australia. The prevalence in these regions is approximately 100–200 cases per 100,000 people [5]. The familial occurrence of MS is relatively low. Genetic studies indicate that genetic predispositions play a significant role [6].

### 1.3. Types of Sexual Dysfunctions

Sexual dysfunctions (SDs) are a broad category of issues that encompass various difficulties related to sexual behaviors, preferences, and experiences. These disorders can affect both physical and emotional aspects, impacting an individual’s life and their relationships with others. Depending on the type of dysfunction, symptoms can take different forms—from a lack of interest in sexual activity to issues with achieving sexual satisfaction to an inability to experience orgasm. Disorders related to sexual trauma also constitute a significant part of sexual difficulties.

SDs can be classified in several different ways depending on the context. The most common way to classify sexual dysfunctions is based on whether they occur in association with gender or independently. Listed below (in Table 1) are the most commonly presented types of sexual dysfunctions reported in the literature, along with brief descriptions. These terms will be used throughout the rest of this review.

The necessity of providing specific care to patients with MS, which significantly differs from working with neurologically unaffected patients, stems from the unique nature of this patient group [9,10]. Sexual dysfunctions in patients with multiple sclerosis can be divided into the following types:

- Primary—resulting from direct damage to the nervous system, which impairs a specific phase of the sexual response to stimulation (sensory disturbances, lubrication issues, erectile dysfunction, pain, orgasm disorders).
- Secondary—a consequence of disease symptoms, which indirectly impair or prevent the fulfillment of sexual functions (fatigue, spasticity, sphincter dysfunction, coordination problems, side effects of treatment).
- Tertiary—emotional, mood, and cognitive function disorders. These are responses to progressive disability, changes in body image, and social roles (low self-esteem, mood disturbances, feelings of guilt, changes in roles performed).

It is important to emphasize that sexual dysfunctions are common problems that can affect anyone, regardless of age, gender, sexual orientation, or the stage of the disease or treatment process.

## 2. Pathogenetic Mechanisms and Prevalence of Sexual Dysfunction in Multiple Sclerosis Patients

When considering the quality of life of patients with MS, the realm of sexuality is often overlooked. Sexual function is a complex process that requires the cooperation of multiple body systems, including the nervous, hormonal, and vascular systems. Damage to the myelin and axons in MS can lead to disruptions in the transmission of nerve signals, directly affecting the ability to experience pleasurable sensations, achieve and maintain erections, feel sexual pleasure, and other aspects of sexual function [11,12,13]. In addition to direct neurological damage, the psychological consequences of living with MS, such as depression, anxiety, and low self-esteem, can further impact sexual function and intimate relationships [14,15]. Many analyses also indicate that patients have significant difficulty in reporting these issues to their physicians. The mechanism behind this phenomenon is not fully understood. Due to the difficulty in finding scientific studies with unequivocal results that were conducted on large groups of participants, it is not possible to fully demonstrate the scale of the problem of sexual dysfunctions. However, the literature provides information that sexual dysfunctions may affect anywhere from 42% to 73% of individuals in this patient group. In 2013, Lew-Starowicz and Rola identified the issue of sexual dysfunction in 73% of women with multiple sclerosis, with the most common problems being related to desire and sexual arousal. A total of 62% of women reported issues with reduced sexual desire. Additionally, broadly defined orgasmic dysfunctions affected 54% of the women studied. Painful intercourse, referred to as dyspareunia, was reported by 35% of women. Importantly, it was found that as disability progressed in the course of the disease, sexual dysfunctions occurred more frequently [16]. A similar epidemiology of sexual dysfunctions in this patient group was demonstrated by Gava and co-authors in their 2019 study [17]. In their review, 63.6% of women with MS experienced sexual dysfunction, with the highest percentage being related to desire disorders (49.3%). This study also proved that women with MS who experience sexual dysfunctions have higher levels of stress and depression, which negatively affect their quality of life. A decrease in the ability to experience orgasm was reported by 41% of participants. It was also noted that patients were less likely to report sexual problems to their doctors, even though the results indicated their high prevalence. Three years later, in the study by Grech and colleagues, men with MS were also included [18]. A total of 68% of men reported difficulties in obtaining or maintaining an erection sufficient for sexual intercourse, making this the most common sexual dysfunction in the male population. Among the most common dysfunctions, researchers have also mentioned sensory disturbances in the genital area, dysesthesia, allodynia, bladder incontinence, and bowel incontinence [16,17,18]. Although sexual dysfunction in this patient population has been the subject of considerable research, standardized diagnostic guidelines for its assessment remain insufficiently defined. Neurophysiological studies have shown that patients with multiple sclerosis (MS) often experience autonomic nervous system dysfunction, including issues related to sexual function. The sympathetic skin response (SSR) may be abnormal in individuals with sexual dysfunction. However, no direct correlation has been found between SSR results and the severity of sexual impairment. Currently, neurophysiological testing is not routinely recommended for diagnosing sexual dysfunction in MS patients. Its use is mainly limited to cases requiring confirmation of peripheral reflex arc damage, for example, in medicolegal contexts. Nevertheless, such testing can provide valuable insights in research on the mechanisms of sexual dysfunction in MS [19].

## 3. Physiotherapy in Multiple Sclerosis-Related Sexual Dysfunction

Sexual dysfunctions related to MS lead to a significant deterioration in patients’ quality of life and negatively impact their social and familial functioning. Such dysfunctions require therapy, just like other neurological deficits associated with MS. Sexual dysfunctions in MS can be treated with pharmacological interventions or through physiotherapeutic and behavioral interventions. Pharmacological interventions were beyond the scope of this review, so we intentionally excluded them. With the advancement of medicine, particularly pelvic floor physiotherapy in recent years, it can be integrated with other aspects of patient treatment. However, it is important to consider the intimacy of the issue that the patient presents. Interventions related to the pelvic area require exceptional sensitivity and attentiveness from specialists. Not every patient will agree to the proposed type of therapy. A key argument in favor of intervention is the fact that many of these methods have demonstrated proven effects in scientific studies for patients. So far, the following physiotherapeutic interventions have been applied (Table 2) [20]:

All the therapies mentioned above can be used in the rehabilitation of sexual dysfunction. This review focused on the following four interventions that have been supported by evidence in clinical studies:

- Pelvic floor exercises [25,26].
- Tibial nerve stimulation [27,28].
- Clitoral vacuum suction/vibration [29].
- Telerehabilitation [30].

## 4. Aim of Review

This review highlights the need to include pelvic floor physiotherapists in MS treatment teams, as multidisciplinary, evidence-based care yields the best outcomes. Physiotherapy can complement pharmacological treatment and improve quality of life. However, data on physiotherapeutic interventions for sexual dysfunctions remain limited due to challenges in conducting clinical trials and accessing reliable sources. The aim of our review is to gather credible data from previously conducted clinical trials and then demonstrate to specialists from various fields the physiotherapeutic options for sexual dysfunctions.

## 5. Methods

We started data collection by searching PubMed and Google Scholar. We used the following research terms: multiple sclerosis, sexual dysfunctions, clinical trials, randomized clinical trials. Excluded from the analysis were review articles, case reports, observational studies without a control group, and opinion articles. In the analysis, we focused on the methods used, the effects of these therapies, group sizes, and the methods of control. We included only clinical trials, randomized clinical trials with a control group receiving a placebo or another form of intervention, and those trials whose results indicated a positive effect on the disorders.

## 6. Results

**Figure 1:**
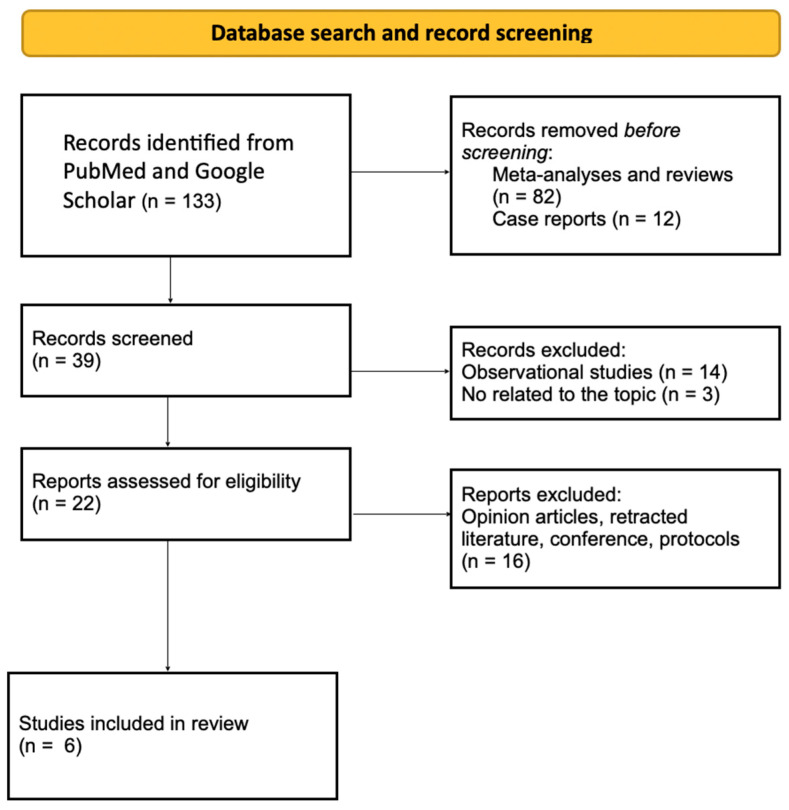
Flow diagram of study selection.

A total of 133 documents were identified through all databases and search methods. After screening the abstracts, every record was assessed for eligibility through a comparison of its full text with the inclusion and exclusion criteria of the study or by consideration of its relevancy. Accordingly, 127 documents were excluded and a total of six articles were included in the systematic review. The detailed description of the excluded studies is presented in the figure below (Figure 1).

### 6.1. Pelvic Floor Muscle Training

For several years, pelvic floor muscle training has been considered the gold standard in the treatment of pelvic floor dysfunction. It is used in conditions related to the urinary system and the lower digestive tract, as well as in sexual disorders.

In the 2018 study by Mosalanejad et al., the authors aimed to assess the impact of pelvic floor muscle exercises combined with mindfulness on sexual function in women with MS [25]. A randomized controlled trial was conducted with participants randomly assigned to one of two groups: pelvic floor exercises with mindfulness, or a control group. In this study, the control group consisted of women with MS who received standard care and were not subjected to pelvic floor muscle exercises or mindfulness interventions. This group allowed the researchers to compare the effects of the combined treatment (pelvic floor muscle exercises and mindfulness) on sexual function against usual care. The intervention lasted for 8 weeks, with two sessions per week. In the experimental group, women performed pelvic floor exercises and participated in mindfulness sessions aimed at stress reduction. A key strength of the study was the use of standard tools to assess sexual function, such as the Female Sexual Function Index (FSFI). Before the intervention, the patients reported low sexual satisfaction and issues with desire and arousal. After the intervention (following six weeks of regular exercises and mindfulness), a statistically significant improvement was observed in sexual satisfaction, particularly in control over sphincter muscles and sensations during intercourse (*p* < 0.05). This improvement also extended to sexual quality of life, indicating improvements in overall satisfaction and in experiencing sexual pleasure. The analysis showed that combining exercises with mindfulness led to better outcomes than isolated pelvic floor therapy. The study also confirmed that mindfulness could be an effective method to support the treatment of sexual dysfunction in women with MS.

In 2014, a study was published to evaluate the effectiveness of pelvic floor muscle training, both in isolation and in combination with electrostimulation, in treating sexual dysfunction in women with MS [26]. The study was a randomized controlled trial in which women were randomly assigned to one of three groups: pelvic floor muscle exercises alone; exercises with electrostimulation; or a control group. This study by Lúcio et al. included two control groups. One group received pelvic floor muscle training alone and the second group received pelvic floor muscle training combined with electrostimulation. These groups were used to evaluate the effectiveness of the two interventions in treating sexual dysfunction in women with MS, so that comparisons could be made between training alone and the combined treatment. The intervention lasted 12 weeks, and the effects were evaluated using the FSFI questionnaire and sexual function measurements. The results indicated a significant improvement in sexual function in both intervention groups, particularly when pelvic floor muscle training was combined with electrostimulation. The electrostimulation group showed greater improvements in sexual arousal and satisfaction, compared with pelvic floor exercises alone. The authors emphasized that electrostimulation could act as a supportive element in therapy, facilitating the effective activation of the pelvic floor muscles.

### 6.2. Tibial Nerve Stimulation

A study published in 2021 aimed to evaluate the impact of transcutaneous tibial nerve stimulation and pelvic floor muscle training on sexual dysfunction in women with MS who reported overactive bladder symptoms [27]. Participants were randomly assigned to one of two groups: electrostimulation; or pelvic floor muscle training. The intervention lasted 12 weeks and outcomes were assessed using questionnaires on sexual function and urological symptoms. Before the intervention, the patients had difficulty with bladder control and reported generally lower sexual satisfaction due to urinary incontinence. After the intervention using TENS and pelvic floor muscle training, a statistically significant improvement in bladder control was observed (*p* < 0.05), along with an increase in sexual satisfaction, particularly in individuals with urinary incontinence issues. Both methods proved effective in improving sexual function, particularly enhancing sexual desire and satisfaction. The electrostimulation group had higher scores for reductions in overactive bladder symptoms, which positively impacted sexual quality of life. Pelvic floor muscle training also resulted in improvements, especially in bladder control. It can thus be inferred that both methods have therapeutic value in treating sexual and urological dysfunctions in women with MS.

In a clinical study published three years later, the effectiveness of transcutaneous tibial nerve stimulation in treating primary sexual dysfunction in MS was investigated [28]. The authors of the study assumed that the tibial nerve arises from the nerve roots that emerge from the lumbar (L4–L5) and sacral (S1–S3) segments of the spinal cord. The tibial nerve is part of the plexus sacralis, from which the pudendal nerve also branches. The pudendal nerve innervates the pelvic floor muscles and is responsible for sensation in the genital and anal regions. Despite the lack of a direct connection, theories have been proposed regarding their indirect influence on each other and the pelvic floor. This was a pilot randomized controlled trial in which participants were randomly assigned to the intervention group (electrostimulation) or the control group. The intervention lasted 12 weeks, with participants undergoing two TENS sessions per week. The stimulation protocol was applied for 30 min to improve sexual function, including libido, orgasm ability, and satisfaction. The results were assessed using sexual function questionnaires such as FSFI and self-reported evaluations from participants. After the therapy, the intervention group showed significant improvements in desire, sexual arousal, and overall sexual satisfaction, compared with the control group. No significant changes in sexual function were observed in the control group. The study concluded that TENS could be a promising method for treating primary sexual dysfunction in patients with MS, improving sexual quality of life. Additionally, the therapy was well tolerated by participants, suggesting its feasibility for use in this patient group.

### 6.3. Clitoral Vacuum Suction/Vibratory Stimulation

Marcalee, Khurram, and others conducted a randomized trial to evaluate the effectiveness of two therapies for the treatment of neurogenic orgasmic dysfunction in women: clitoral vacuum suction and vibratory stimulation [29]. In this study, orgasmic dysfunction was defined as difficulty in achieving orgasm due to neurogenic damage. Specifically, it referred to women with neurogenic orgasmic dysfunction (NOD). The study was a randomized controlled trial, with participants randomly assigned to one of two groups. In the experimental group, women used clitoral vacuum suction devices while the other group used vibratory stimulation. The goal of the intervention was to improve orgasmic ability. Effectiveness was evaluated using questionnaires and self-reports. The results indicated that both methods were effective in improving orgasm; however, vacuum suction was slightly more effective in enhancing orgasm intensity. The authors assessed orgasm dysfunction in participants using a declarative methodology, based on subjective reports from the patients. The patients were asked about their experiences related to achieving orgasm, as well as the quality of their sexual experiences, which allowed for the evaluation of the impact of the intervention on the problem of NOD. Proprietary questionnaires were also used in which participants self-assessed their sexual functions, including difficulties in achieving orgasm. Such an approach is characteristic of research in sexual dysfunctions, as it is difficult to objectively measure subjective experiences such as orgasm. As a result, both methods were well tolerated by the participants. The study suggests promising applications of these technologies in the treatment of sexual dysfunctions in women with neurogenic sexual problems.

### 6.4. Telerehabilitation

The final methods analyzed in this review were telerehabilitation approaches for treating sexual dysfunction in MS. A comparison of three different telerehabilitation protocols was presented in a study published in *Neurological Sciences* in 2024 [30]. The aim of this study was to compare different telerehabilitation protocols for managing urogenital symptoms in women with MS. Participants were randomly assigned to one of three groups based on the type of therapy: rehabilitation through videoconferencing; self-exercises with a mobile app; or standard in-person rehabilitation. The interventions lasted for 8 weeks, and the outcomes were evaluated using questionnaires on bladder function, quality of life, and sexual symptoms. It was found that telerehabilitation was effective in reducing urogenital symptoms, with similar results being obtained for in-person and remote rehabilitation. The best results were achieved in the group that used a mobile app in combination with individual online sessions. Telerehabilitation proved to be a convenient and effective solution, especially during the COVID-19 pandemic. These findings suggest that telerehabilitation could be a valuable method for managing urogenital symptoms in women with MS.

The main findings of the studies included in this review are presented in the Table 3 below.

## 7. Discussion

To the best of our knowledge, this is the first review that comprehensively examines physiotherapeutic interventions for sexual dysfunctions in patients with multiple sclerosis (MS). Although the number of randomized clinical trials (RCTs) in this area remains limited, the available studies provide promising evidence of the effectiveness of various physiotherapeutic methods. Across the six RCTs included, a range of interventions—such as pelvic floor muscle training, tibial nerve stimulation (TNS), clitoral vacuum/vibration therapy, and telerehabilitation—were used, often in combination with other techniques like mindfulness. The consistent finding among these studies is the improvement in sexual function and quality of life, particularly among women, who were the primary focus of most trials. Improvements were most often observed in parameters such as desire, arousal, orgasmic function, and control over sphincter muscles. Importantly, the combination of pelvic floor training with mindfulness techniques appears to offer enhanced benefits, suggesting that physiotherapy targeting both physical and psychological dimensions may be more effective than purely somatic approaches. This integrative strategy aligns with the broader understanding of sexual dysfunction in MS as multifactorial, influenced by both neurogenic and psychosocial factors. The heterogeneity of interventions and assessment tools, including the use of the FSFI questionnaire and urological symptom scales, indicates a need for more standardized protocols. Moreover, while outcomes were generally positive, sample sizes were small, and few studies included men, leaving an important gap in the literature. Overall, the findings underscore the potential of physiotherapy as a supportive treatment in managing sexual dysfunction in MS. However, further research is needed to clarify the most effective intervention types, determine optimal treatment parameters, and assess long-term outcomes across diverse patient populations. The inclusion of pelvic floor physiotherapists in multidisciplinary teams is consistently recommended and may represent a key step toward more holistic and personalized care. Based on the analyzed studies and existing literature in this field, it appears reasonable to pursue further research that includes the following directions:

- expanding research on the effectiveness of specific physiotherapeutic methods in the context of sexual dysfunction in MS patients, with larger study populations and longer follow-up periods;
- determining the optimal parameters of therapy (frequency, intensity, duration) that yield the best outcomes in improving sexual function and patients’ quality of life;
- investigating the effectiveness of a personalized therapeutic approach that includes interdisciplinary collaboration—particularly involving pelvic floor physiotherapists—as part of comprehensive patient care.

## 8. Conclusions

In conclusion, there are numerous evidence-based physiotherapeutic methods that can effectively complement the treatment of patients with multiple sclerosis who experience sexual dysfunctions. All analyzed types of physiotherapeutic treatment have all been shown to positively impact sexual quality of life, addressing key issues such as desire, arousal, and satisfaction. These therapies are particularly beneficial when integrated into a comprehensive treatment plan. However, the data collected so far still require further research. Further studies with larger sample sizes are also required, along with broader explorations of different physiotherapeutic approaches, to confirm their long-term effectiveness. More studies are also needed to determine the specific parameters for optimal outcomes, and to explore the potential for personalized care. By recognizing the importance of pelvic floor therapy, we can ensure more targeted and holistic interventions—leading to better patient outcomes and higher standards of care.

## Figures and Tables

**Table 1:** Sexual Dysfunctions.

No.	Name	Sex It Concerns	Meaning
1.	Sexual desire disorders	Male and Female	Decreased interest or lack of interest in sex; lack of sexual initiative and desire for sexual stimulation [7,8].
2.	Sexual arousal disorders	Female	Difficulties in achieving or maintaining an adequate level of sexual arousal, such as problems with vaginal lubrication [7].
3.	Orgasm disorders	Male and Female	Difficulties in achieving orgasm despite appropriate arousal; delayed or absent orgasm; in man often related to delayed ejaculation [7,8].
4.	Dyspareunia	Male and Female	Pain during penetration (female); pain in the genital area during sexual activity (male) [7,8].
5.	Vaginismus	Female	Tension and muscle spasm of the vagina that prevent penetration [7].
6.	Erectile dysfunctions	Male	Difficulties in achieving or maintaining an erection sufficient for penetration [8].
7.	Psychological and emotional disorders	Male and Female	Anxiety, stress, depression, and mood disorders that can affect sexual response [7,8].
8.	Medication-induced sexual dysfunction	Male and Female	Medications such as antidepressants, antihypertensive drugs, or painkillers can cause changes in sexual functioning [7,8].
9.	Disorders resulting from interpersonal issues	Male and Female	Relationship problems, difficulties in communication with a partner, sexual misunderstanding [7,8].

**Table 2:** Physiotherapeutic interventions in SDs.

No.	Name	Explanation
1.	Individual and group pelvic floor muscle training [21]	Regular exercises can improve control over sphincters and influence blood circulation in the pelvis, which can positively affect sensations during intercourse.
2.	Manual therapy [21]	Restoring proper muscle tension in the pelvic region can improve comfort during sexual activity and help reduce discomfort caused by spasticity.
3.	Thermal therapy (heat and cold) [21]	Thermal stimulus can help reduce muscle tension and improve blood circulation, which can positively influence sexual function especially in cases involving pain.
4.	Breathing exercises [22]	Breathing techniques are used to improve overall well-being, reduce stress, and enhance the sensation of sexual pleasure.
5.	Electrostimulation [23]	Involves using electrical impulses to simulate the pelvic muscles. It is applied in the treatment of erectile dysfunction in men, as well as in the treatment of urinary incontinence and enhancement of sexual sensations in women.
6.	Biofeedback [23]	Specifically tailored pelvic floor muscle training combined with visual stimulation influences sensory perception of the deep muscle layers in the pelvis, thereby improve sexual sensations.
7.	Behavioral therapy [24]	May include discussions about intimacy and relationships, as well as relaxation techniques combined with physical exercises.

**Table 3:** Main findings of six articles included in the current study.

No.	First Author	Year of Publication	Sample Size	Control Group	Intervention Effect
1.	Mosalanejad Fatemeh [25]	2018	60 women with MS and sexual dysfunction.	30 women without PF exercises.	The combination of pelvic floor muscle exercises and mindfulness techniques had a positive impact on the participants’ sexual function, increasing sexual satisfaction and reducing sexual dysfunction.
2.	Lúcio AC [26]	2014	45 women with MS and sexual dysfunction.	Groups of 15 women were assigned to: pelvic floor muscle exercises; electrostimulation; or a combination of both methods.	Both forms of intervention were effective in improving sexual function, with greater benefits observed in the group combining electrostimulation.
3.	Giannopapas Vasileios [27]	2024	40 patients with MS.	20 patients without tibial nerve stimulation.	Tibial nerve stimulation proved to have a positive impact on sexual dysfunction.
4.	Polat Dunya Cansu [28]	2021	75 women with MS, overactive bladder and sexual dysfunction.	Groups of 25 women were assigned to: pelvic floor training; tibial nerve stimulation; or without any therapy.	Both methods improved sexual function.
5.	Alexander Marcalee [29]	2018	90 women with neurogenic orgasmic dysfunction.	Groups of 30 women were assigned to: vacuum suction; vibratory stimulation; or no stimulation (placebo).	Both interventions were effective in treating orgasmic dysfunction, with comparable results in both groups.
6.	Deodato Manuela [30]	2024	120 women with MS and urogenital symptoms.	Groups of 40 women were assigned to: rehabilitation through videoconferencing; self-exercises with a mobile app; or standard in-person rehabilitation.	Telerehabilitation may be an effective method for treating urogenital symptoms in women with MS.
